# Supplementary material for: Grand canonically optimized grain boundary phases in hexagonal close-packed titanium
Source: Nat Commun. 2024 Aug 15;15:7049. doi: 10.1038/s41467-024-51330-9 (PMC11327258; doi:10.1038/s41467-024-51330-9)
Supplement: Supplementary file 1 — Supplementary Information [file 41467_2024_51330_MOESM1_ESM.pdf]

Supplementary Information for
Grand canonically optimized grain boundary phases in hexagonal close-packed titanium

Enze Chen[1,2,3,4,*], Tae Wook Heo[2], Brandon C. Wood[2], Mark Asta[1,3], Timofey Frolov[2,†]

[1] Department of Materials Science and Engineering, University of California, Berkeley, CA 94720, USA

[2] Materials Science Division, Lawrence Livermore National Laboratory, Livermore, CA 94550, USA

[3] Materials Sciences Division, Lawrence Berkeley National Laboratory, Berkeley, CA 94720, USA

[4] Present address: Department of Materials Science and Engineering, Stanford University, Stanford, CA 94305, USA

Email: *enze@stanford.edu; †frolov2@llnl.gov

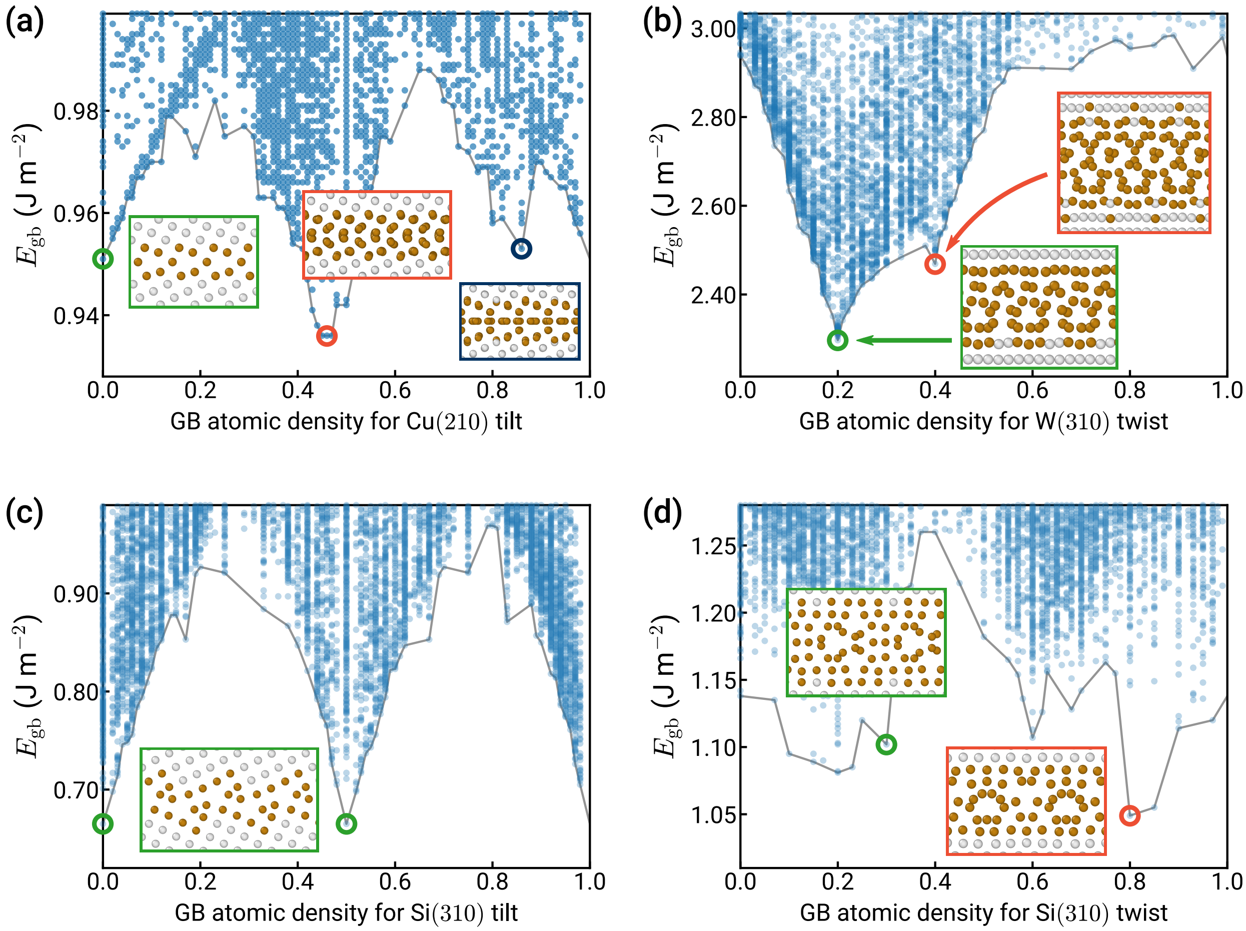

Supplementary Figure 1: Validation of the Grand canonical Interface Predictor (GRIP) tool. We perform grand canonical optimization of the (a) Σ5(210)[001] tilt grain boundary (GB) in Cu (using an embedded-atom method (EAM) potential[1]), (b) Σ5(310)[001] twist GB in W (EAM[2]), (c) Σ5(310)[001] tilt and (d) Σ5(310)[001] twist GBs in Si (Stillinger-Weber[3]). The energy vs. density plots and low-energy structures match those in the literature for Cu,[4] W,[5] and Si.[6,7] The atoms in the inset structures are colored according to the common neighbor analysis,[8] where gray are bulk-coordinated atoms and brown are non-bulk-coordinated atoms (in the GB).

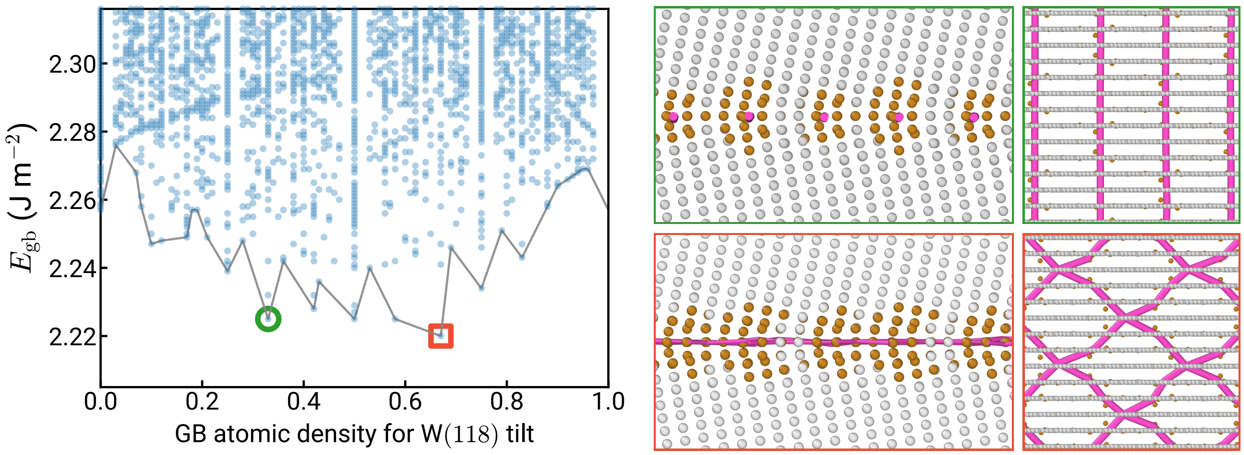

Supplementary Figure 2: Discovering a new ground state in W$(118)[1\bar{1}0]$. A previous study[5] using the evolutionary algorithm USPEX[9,10] and an embedded-atom method (EAM) potential[2] found a ground state for the $\mathrm{W}(118)[1\bar{1}0]$ grain boundary (GB) at $[n] = 0.33$ with GB energy $E_{\mathrm{gb}} = 2.225\,\mathrm{J\,m^{-2}}$ (green circle), as pictured in the top two panels. Using the Grand canonical Interface Predictor[11] and the same EAM potential, we find a structure with lower energy $E_{\mathrm{gb}} = 2.220\,\mathrm{J\,m^{-2}}$ at $[n] = 0.67$ (orange square), as shown in the bottom two panels. This structure has a different dislocation network where the $\langle 001 \rangle$-type edge dislocations (magenta, as identified using the dislocation extraction algorithm[12]) overlap in the GB plane instead of residing in parallel.

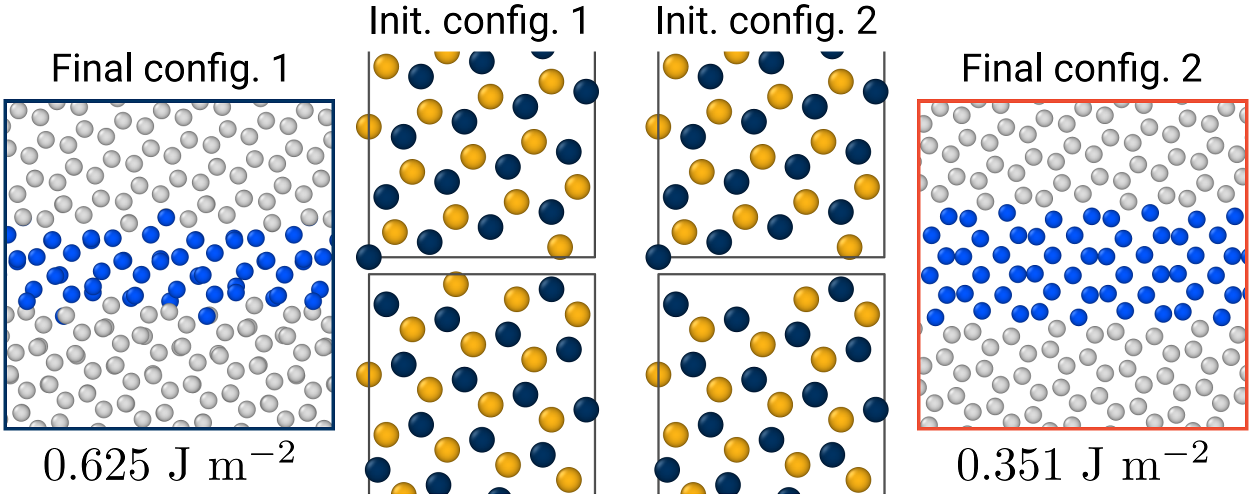

Supplementary Figure 3: Different planar terminations and corresponding grain boundary (GB) structures. $\{31\bar{4}0\}[0001]$ is a GB where the two basis atoms have different z coordinates normal to the GB plane, and two initial configurations are shown where the lower slabs terminate at different basis atoms (gold for the first, blue for the second). We use the γ-surface method to optimize the GB structure in both cases. The first configuration ($[n] = 0$) produces a higher-energy structure shown on the left ($E_{\rm gb} = 0.625\,\mathrm{J\,m^{-2}}$), while the second configuration ($[n] = 0.5$) produces the lower-energy structure shown on the right ($E_{\rm gb} = 0.351\,\mathrm{J\,m^{-2}}$), which matches the ground-state structure from the Grand canonical Interface Predictor. The final structures and colors correspond to those in Fig. 3 in the main manuscript.

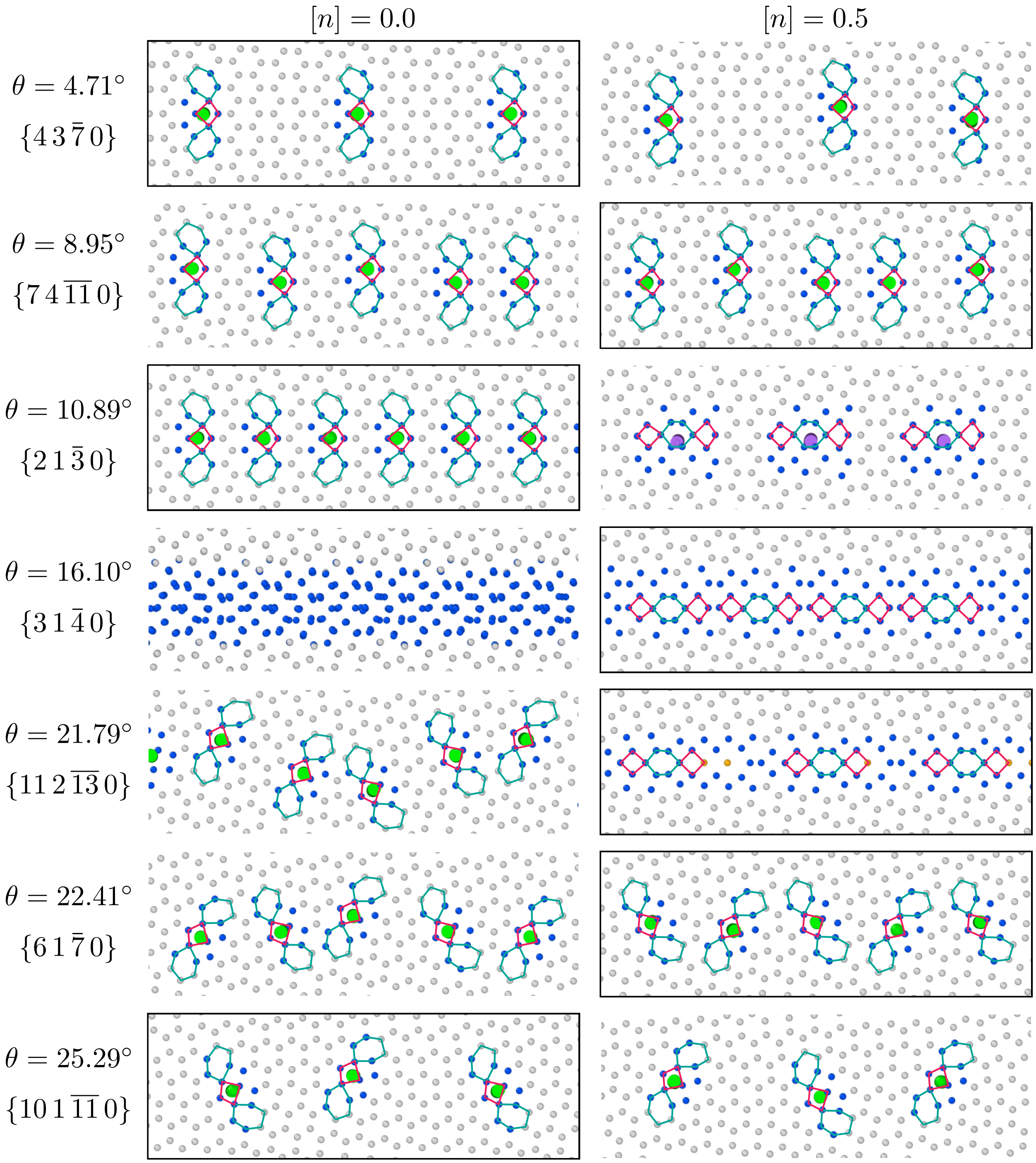

Supplementary Figure 4: Grain boundary (GB) structural units in $[0001]$ **symmetric tilt GBs**. Results for select GBs at $[n] = 0.0$ and $[n] = 0.5$ are viewed down $[0001]$. Low-angle GBs adopt a dislocation core configuration at $[n] = 0.0$ that is accommodated at $[n] = 0.5$ through dislocation climb. At a tilt angle of $\theta \approx 10.89^\circ$, there is a transition at $[n] = 0.5$ to a different structural unit. At even higher angles $\theta \geq 22.41^\circ$, the GB structural units transform back into the motifs at low angles. The lower-energy structure at each tilt angle, as evaluated using the modified embedded-atom method (MEAM) potential,[13] is outlined in black.

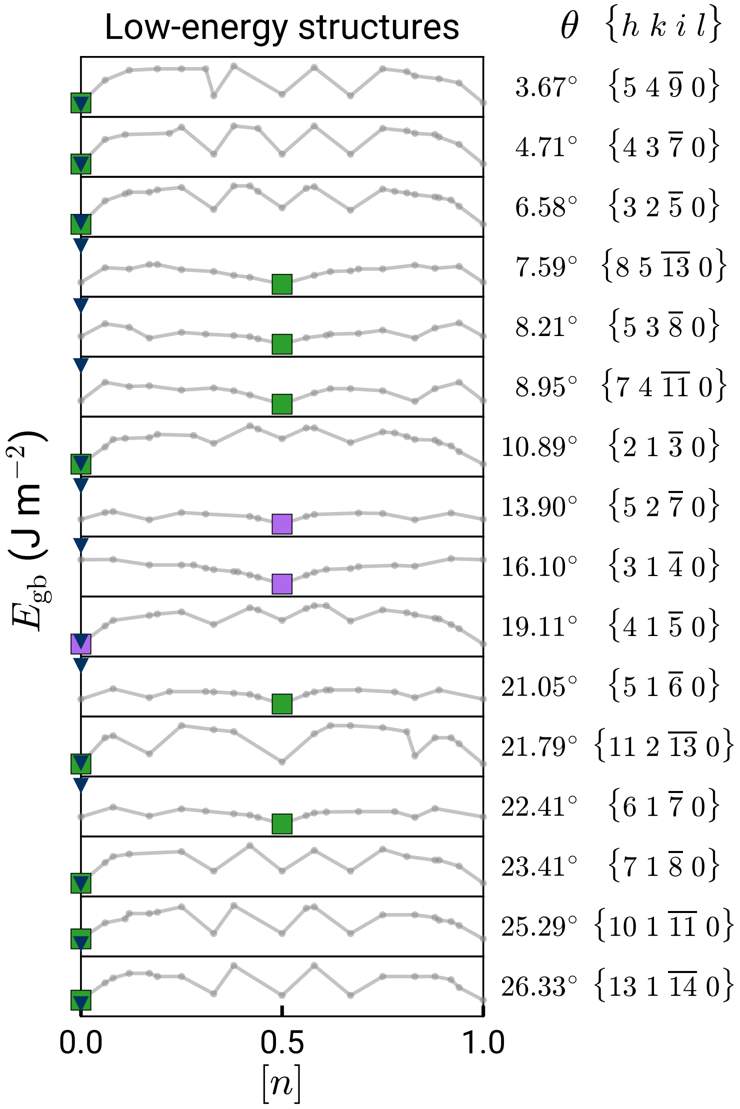

Supplementary Figure 5: Energy map of [0001] **grain boundaries simulated using an embedded-atom method (EAM) potential.**[14] The profiles are qualitatively similar to those in Fig. 4 in the main text, which was generated using a modified embedded-atom method (MEAM) potential.[13] See Fig. 4 for additional descriptions of features.

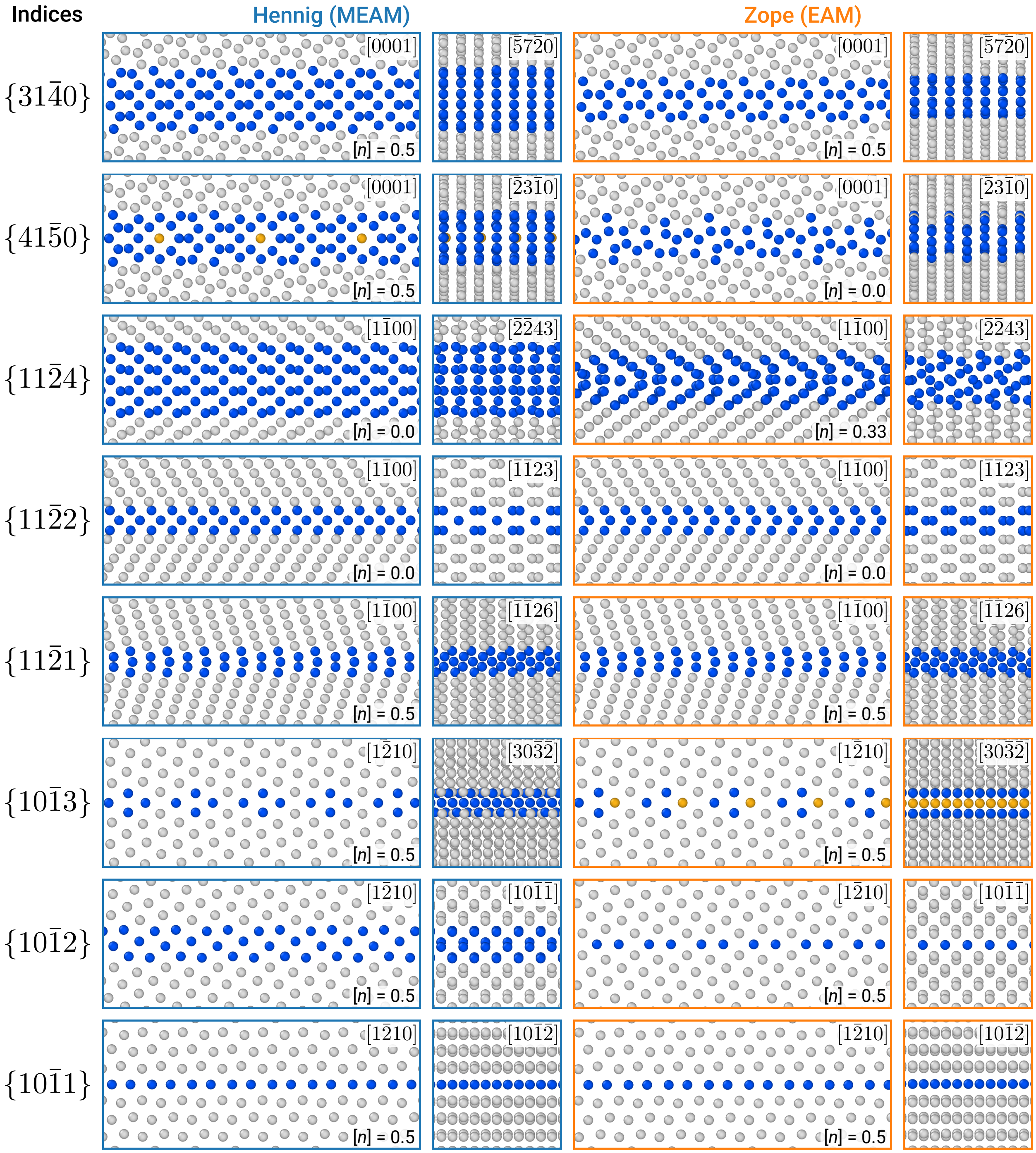

Supplementary Figure 6: Comparison of optimized grain boundary (GB) structures. A few symmetric tilt GBs are provided for each tilt axis and two projections are shown for each ground state obtained using the modified embedded-atom method (MEAM)[13] and embedded-atom method (EAM)[14] potentials. The optimal GB atomic density ([n]) is shown in the lower-right corner and may not be equal for both potentials. The atoms are colored according to the common neighbor analysis in OVITO,[8,15] where gray are hexagonal close-packed-coordinated atoms, gold are face-centered cubic-coordinated atoms, and blue have a different coordination (in the GB).

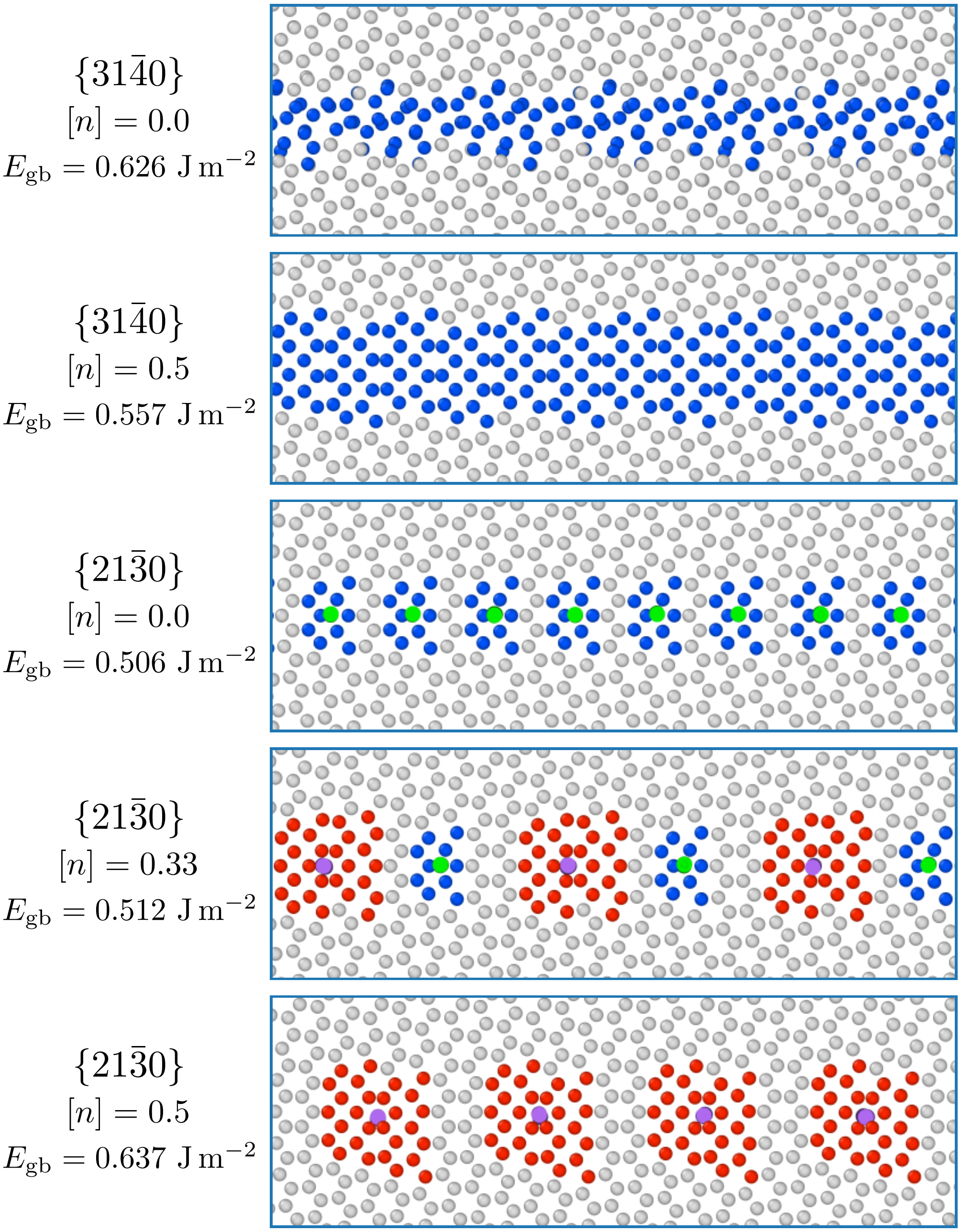

Supplementary Figure 7: Density functional theory validation for select grain boundaries (GBs). The structures from the Grand canonical Interface Predictor (GRIP) are used as inputs to VASP[16–19] for further relaxation (see Methods in the main manuscript for details). The GB dislocation core structures (highlighted with colored atoms) remain stable and the relative energies between different phases are consistent with those from GRIP.

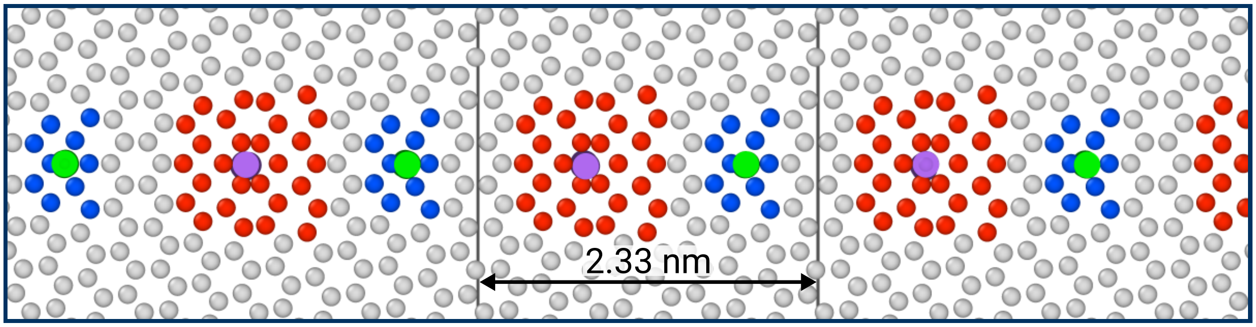

Supplementary Figure 8: Mixed state at $[n] = 0.33$ **and** $[n] = 0.67$ **for** $\{21\bar{3}0\}[0001]$. At intermediate values of $[n] \in (0, 0.5)$, the localized dislocation cores in the grain boundary alternate between $\mathbf{b}_\mathrm{I} = \frac{1}{3}\langle 1\bar{2}10\rangle$ (green) and $\mathbf{b}_\mathrm{II} = \frac{1}{3}\langle 2\bar{4}20\rangle$ (purple) dislocations to obtain the minimum-energy structure. The amount of each phase follows the conventional lever rule for phase fractions. Dislocations are identified using the dislocation extraction algorithm in OVITO.[12,15]

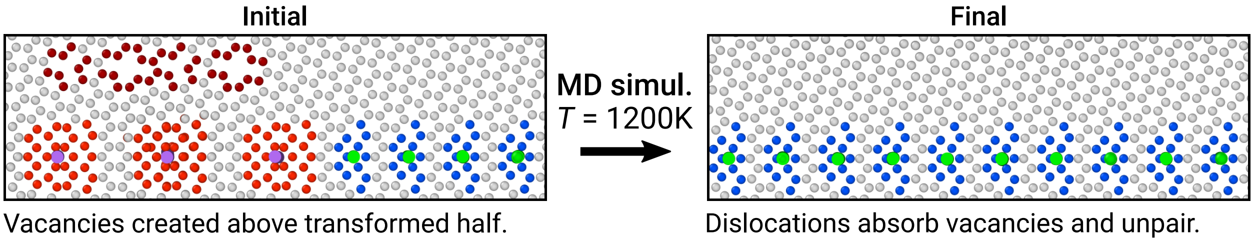

Supplementary Figure 9: (Reverse) phase transformation through vacancy absorption. In the main text, we demonstrate interstitial-induced phase transformation and coexistence in $\{21\bar{3}0\}[0001]$, where every two $\mathbf{b}_\mathrm{I}$ dislocations pair up to form one $\mathbf{b}_\mathrm{II}$ dislocation. Here, by injecting vacancies (outlined in dark red) and performing molecular dynamics (MD) simulations at $T = 1200\,\mathrm{K}$, we reverse the transformation, whereby the $\mathbf{b}_\mathrm{II}$ dislocations absorb the vacancies and unpair.

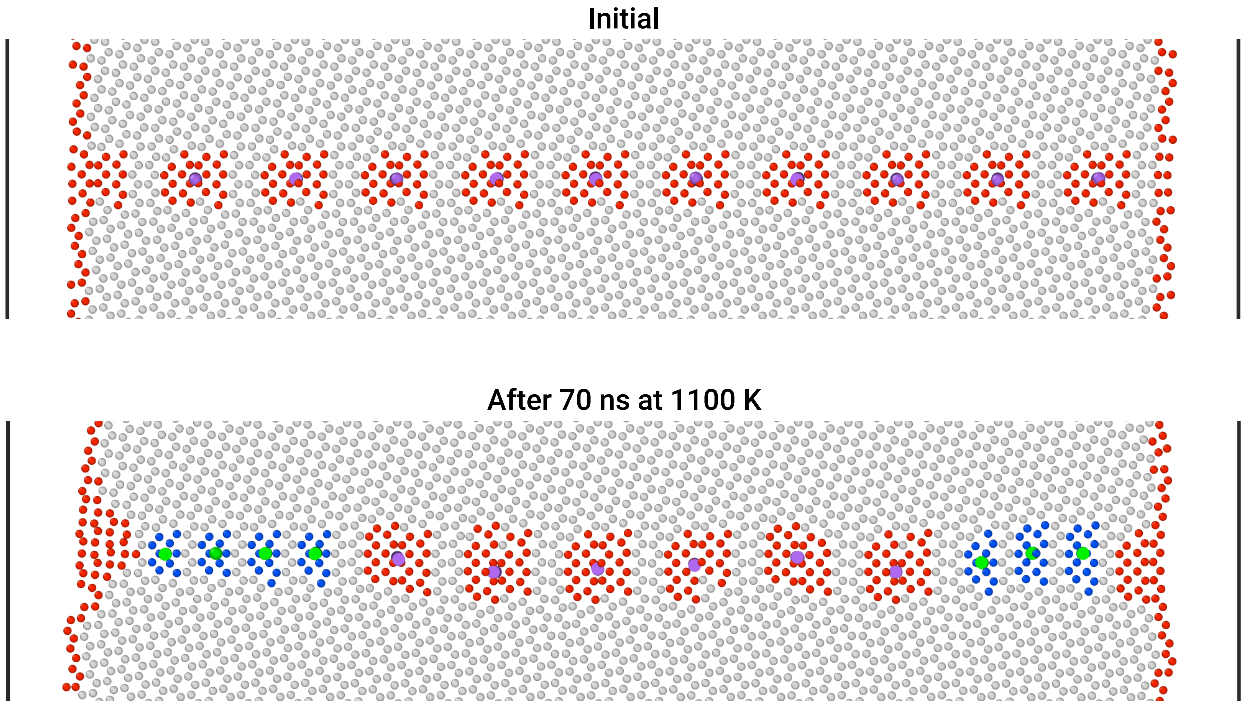

Supplementary Figure 10: Open-surface molecular dynamics simulations show a gradual transformation of the $\{21\bar{3}0\}[0001]$ **grain boundary (GB)**. Starting from the metastable $[n] = 0.5$ state (highlighted with red atoms), the GB transforms to the $[n] = 0.0$ state (blue atoms) as atoms diffuse in from the surface. We emphasize that this open-surface simulation method is not a robust GB structure prediction method for ground-state and low-temperature phases, as discussed in the main text.

Supplementary Note 1

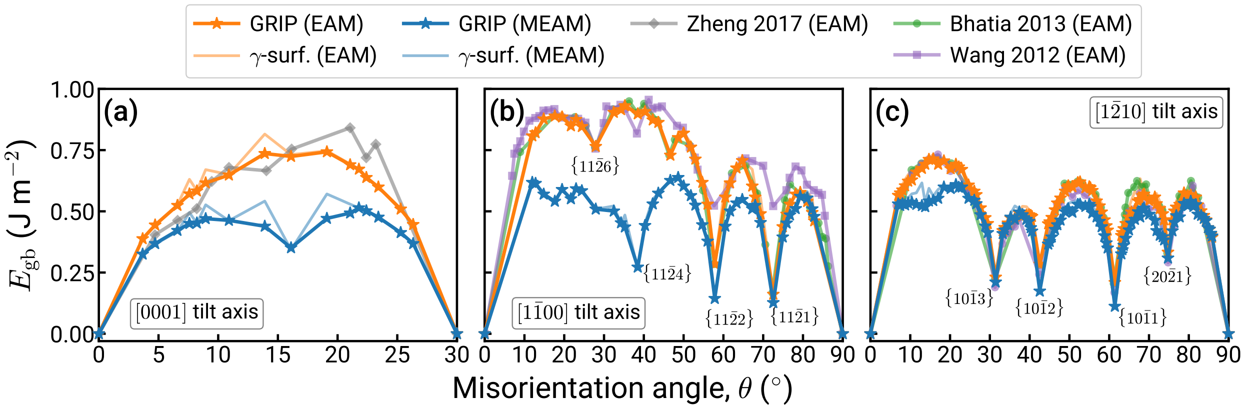

Supplementary Figure 11: Energy vs. tilt angle for symmetric tilt grain boundaries in α-Ti. All results are shown for the (a) [0001], (b) [1$\bar{1}$00], and (c) [1$\bar{2}$10] tilt axes. Each point corresponds to the minimum-energy structure for that tilt angle. The orange and blue lines correspond to our calculations using the embedded-atom method (EAM)[14] and modified embedded-atom method (MEAM)[13] potentials, respectively. Solid lines with stars are for the Grand canonical Interface Predictor (GRIP) and translucent lines are for the γ-surface method. The other data (gray in (a) and green/purple in (b) and (c)) are referenced from the literature.[20–23] Select twin boundaries corresponding to energy cusps are labeled in panels (b) and (c).

To better characterize where our Grand canonical Interface Predictor (GRIP) algorithm improves upon existing studies, we compare plots of grain boundary energy (E_{gb}) vs. the tilt angle (θ) for all ground-state structures in Supplementary Fig. 11. Using the embedded-atom method (EAM) potential from Zope and Mishin[14] (solid orange), we match or improve upon the results from Bhatia and Solanki[21] (green) and Wang and Beyerlein[22,23] (purple) for the [1$\bar{1}$00] and [1$\bar{2}$10] symmetric tilt grain boundaries (STGBs), as seen in panels (b) and (c). We note in panel (b) that the results from Wang and Beyerlein[23] exhibit discrepancies in E_{gb} compared to our calculations for certain orientations ({11$\bar{2}$4}, {11$\bar{2}$2}, and {11$\bar{2}$1}), whereas the results reported by Bhatia and Solanki[21] show excellent agreement. At present, the origin of these discrepancies remains unclear. Our results for [0001] STGBs are in good agreement with those from Zheng, et al.[20] (gray), although more precise comparisons are not possible as they used a different EAM potential.[24] We also perform γ-surface calculations sampling all terminations for each STGB and plot the results in corresponding translucent colors in Supplementary Fig. 11. For both the EAM (orange) and MEAM (blue) potentials, the GRIP data (solid lines) are lower bounds for the γ-surface values, which is consistent with expectations. The majority of the discrepancies between the GRIP and

γ-surface data occur for the family of [0001] STGBs. We find for the other two tilt axes closer agreement for E_{gb}, even when many STGBs require grand canonical optimization, as seen in Fig. 5 in the main text. Consistent with the only existing study that used an evolutionary algorithm to study a few GBs in a hexagonal close-packed (HCP) metal (Mg),[25] we also observe a zigzag distribution of GB dislocations instead of a straight line in several optimized $[1\bar{1}00]$ and $[1\bar{2}10]$ STGBs. As it appears that GB phases are more prevalent for certain tilt axes than others, the high-throughput exploration of different materials and boundary types enabled by GRIP would allow us to investigate how bicrystallography affects the phase behavior of GBs.

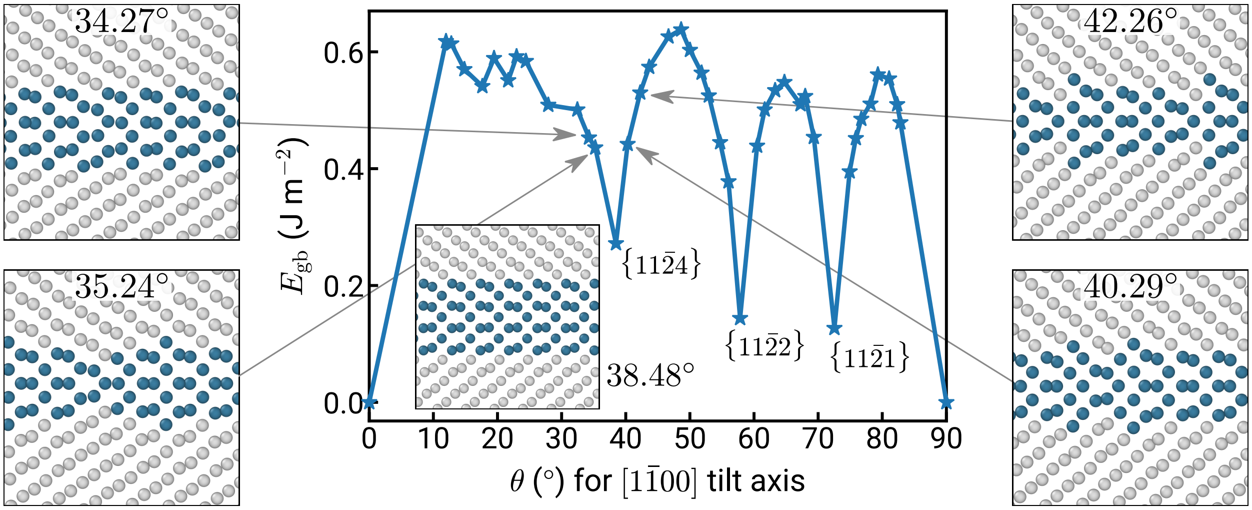

Supplementary Figure 12: Faceting of the boundary in $[1\bar{1}00]$ boundaries. The $\{11\bar{2}4\}[1\bar{1}00]$ ($\theta \approx 38.5°$) twin boundary when simulated with the modified embedded-atom method potential adopts a thick interfacial structure that is a strained version of a metastable bulk polymorph.[26,27] At nearby misorientation angles, the interfacial phase is partly preserved and the boundaries with the surrounding α-Ti slabs are faceted.

Different empirical potential formalisms are expected to result in different GB properties, but previous benchmark studies on STGBs in cubic metals[28] and HCP α-Zr[29] found largely similar E_{gb} vs. θ profiles using the γ-surface method. In contrast, we find notable differences for multiple tilt axes in α-Ti when comparing the EAM[14] (orange) and MEAM[13] (blue) parameterizations. In the family of [0001] STGBs, the values for E_{gb} from the MEAM potential are consistently lower than those produced by the EAM potential, with an energy cusp (local minimum) at $\theta \approx 16.1°$. Likewise, in the family of $[1\bar{1}00]$ STGBs (Supplementary Fig. 11b), only using the MEAM potential do we recover a low-energy $\{11\bar{2}4\}$ twin boundary at $\theta \approx 38.5°$ that we have extensively characterized

using transmission electron microscopy and density functional theory calculations.[26] There, we found the twin boundary to adopt a thick body-centered orthorhombic (BCO) structure that is a strained version of a metastable bulk polymorph of Ti;[27] however, what this high-throughput study also reveals is faceting around the interfacial phase at nearby tilt angles to accommodate the strained BCO phase, as shown in Supplementary Fig. 12. Stabilization of the BCO phase may be responsible for the significantly lower energy of $[1\bar{1}00]$ STGBs simulated with the MEAM potential vs. the EAM potential shown in Supplementary Fig. 11b.

References

[1] Y. Mishin, M. J. Mehl, D. A. Papaconstantopoulos, A. F. Voter, and J. D. Kress. Structural stability and lattice defects in copper: *Ab initio*, tight-binding, and embedded-atom calculations. *Phys. Rev. B.* **63**, 224106 (2001).

[2] X. Zhou, R. A. Johnson, and H. N. G. Wadley. Misfit-energy-increasing dislocations in vapor-deposited CoFe/NiFe multilayers. *Phys. Rev. B.* **69**, 144113 (2004).

[3] F. H. Stillinger and T. A. Weber. Computer simulation of local order in condensed phases of silicon. *Phys. Rev. B.* **31**, 5262–5271 (1985).

[4] T. Frolov, D. L. Olmsted, M. Asta, and Y. Mishin. Structural phase transformations in metallic grain boundaries. *Nat. Commun.* **4**, 1–7 (2013).

[5] T. Frolov, Q. Zhu, T. Oppelstrup, J. Marian, and R. E. Rudd. Structures and transitions in bcc tungsten grain boundaries and their role in the absorption of point defects. *Acta Mater.* **159**, 123–134 (2018).

[6] A. D. Banadaki, M. A. Tschopp, and S. Patala. An efficient Monte Carlo algorithm for determining the minimum energy structures of metallic grain boundaries. *Comput. Mater. Sci.* **155**, 466–475 (2018).

[7] S. von Alfthan, P. D. Haynes, K. Kaski, and A. P. Sutton. Are the structures of twist grain boundaries in silicon ordered at 0 K? *Phys. Rev. Lett.* **96**, 055505 (2006).

[8] A. Stukowski. Structure identification methods for atomistic simulations of crystalline materials. *Model. Simul. Mater. Sci. Eng.* **20**, 045021 (2012).

[9] A. R. Oganov and C. W. Glass. Crystal structure prediction using *ab initio* evolutionary techniques: Principles and applications. *J. Chem. Phys.* **124**, 244704 (2006).

[10] A. O. Lyakhov, A. R. Oganov, H. T. Stokes, and Q. Zhu. New developments in evolutionary structure prediction algorithm USPEX. *Comput. Phys. Commun.* **184**, 1172–1182 (2013).

[11] E. Chen, T. W. Heo, B. C. Wood, M. Asta, and T. Frolov. Grand canonically optimized grain boundary phases in hexagonal close-packed titanium. *GRIP* `https://doi.org/10.5281/zenodo.12590125`, (2024).

[12] A. Stukowski, V. V. Bulatov, and A. Arsenlis. Automated identification and indexing of dislocations in crystal interfaces. *Model. Simul. Mater. Sci. Eng.* **20**, 085007 (2012).

[13] R. G. Hennig, T. J. Lenosky, D. R. Trinkle, S. P. Rudin, and J. W. Wilkins. Classical potential describes martensitic phase transformations between the α, β, and ω titanium phases. *Phys. Rev. B.* **78**, 054121 (2008).

[14] R. R. Zope and Y. Mishin. Interatomic potentials for atomistic simulations of the Ti–Al system. *Phys. Rev. B.* **68**, 024102 (2003).

[15] A. Stukowski. Visualization and analysis of atomistic simulation data with OVITO—the Open Visualization Tool. *Model. Simul. Mater. Sci. Eng.* **18**, 015012 (2009).

[16] G. Kresse and J. Hafner. *Ab initio* molecular dynamics for liquid metals. *Phys. Rev. B.* **47**, 558–561 (1993).

[17] G. Kresse and J. Hafner. *Ab initio* molecular-dynamics simulation of the liquid-metal–amorphous-semiconductor transition in germanium. *Phys. Rev. B.* **49**, 14251–14269 (1994).

[18] G. Kresse and J. Furthmüller. Efficient iterative schemes for *ab initio* total-energy calculations using a plane-wave basis set. *Phys. Rev. B.* **54**, 11169–11186 (1996).

[19] G. Kresse and J. Furthmüller. Efficiency of *ab initio* total energy calculations for metals and semiconductors using a plane-wave basis set. *Comput. Mater. Sci.* **6**, 15–50 (1996).

[20] Z. Zheng, D. S. Balint, and F. P. E. Dunne. Investigation of slip transfer across HCP grain boundaries with application to cold dwell facet fatigue. *Acta Mater.* **127**, 43–53 (2017).

[21] M. A. Bhatia and K. N. Solanki. Energetics of vacancy segregation to symmetric tilt grain boundaries in hexagonal closed pack materials. *J. Appl. Phys.* **114**, 244309 (2013).

[22] J. Wang and I. J. Beyerlein. Atomic structures of symmetric tilt grain boundaries in hexagonal close packed (hcp) crystals. *Model. Simul. Mater. Sci. Eng.* **20**, 024002 (2012).

[23] J. Wang and I. J. Beyerlein. Atomic structures of $[0\bar{1}10]$ symmetric tilt grain boundaries in hexagonal close-packed (hcp) crystals. *Metall. Mater. Trans. A* **43**, 3556–3569 (2012).

[24] G. J. Ackland. Theoretical study of titanium surfaces and defects with a new many-body potential. *Philos. Mag. A* **66**, 917–932 (1992).

[25] C. Yang, M. Zhang, and L. Qi. Grain boundary structure search by using an evolutionary algorithm with effective mutation methods. *Comput. Mater. Sci.* **184**, 109812 (2020).

[26] M. S. Hooshmand, R. Zhang, Y. Chong, et al. Twin-boundary structural phase transitions in elemental titanium. *arXiv:2103.06194* (2021).

[27] N. A. Zarkevich and D. D. Johnson. Titanium α–ω phase transformation pathway and a predicted metastable structure. *Phys. Rev. B.* **93**, 020104 (2016).

[28] B. Waters, D. S. Karls, I. Nikiforov, et al. Automated determination of grain boundary energy and potential-dependence using the OpenKIM framework. *Comput. Mater. Sci.* **220**, 112057 (2023).

[29] E. Torres. Atomistic study of the structure and deformation behavior of symmetric tilt grain boundaries in α-zirconium. *Comput. Mater. Sci.* **197**, 110600 (2021).
